# Incomplete Tests of Conditional Association for the Assessment of Model Assumptions

**DOI:** 10.1007/s11336-022-09841-1

**Published:** 2022-02-05

**Authors:** Rudy Ligtvoet

**Affiliations:** Department Erziehungs- und Sozialwissenschaften, University of Cologne, Germany, Gronewaldstr. 2a, 50931 Cologne, Deutschland

**Keywords:** Conditional association, manifest monotonicity, model complexity, monotone homogeneity model, monotone likelihood ratio, multivariate totally positive of order 2, nonnegative partial correlations, scalability coefficient, strongly positive orthant dependency

## Abstract

Many of the models that have been proposed for response data share the assumptions that define the *monotone homogeneity* (MH) model. Observable properties that are implied by the MH model allow for these assumptions to be tested. For binary response data, the most restrictive of these properties is called *conditional association* (CA). All the other properties considered can be considered incomplete tests of CA that alleviate the practical limitations encountered when assessing the MH model assumptions using CA. It is found that the assessment of the MH model assumptions with an incomplete test of CA, rather than CA, is generally associated with a substantial loss of information. We also look at the sensitivity of the observable properties to model violation and discuss the implications of the results. It is argued that more research is required about the extent to which the assumptions and the model specifications influence the inferences made from response data.

In educational and psychological testing, latent variable models are used to account for the dependencies between the responses to multiple test items, where no one item by itself accurately represents the attribute that the test is supposed to measure. The purpose of the model is to provide an estimate of the latent variable, based on the observed responses to the test items. Many different latent variable models are used in practice, each with their own particular set of assumptions, and applicable to different type of inferences. For example, the *unidimensional* (UD) Rasch (1960) allows for the calibration of all respondents on a common linear scale (Kelderman, 1988; Wright, 1977), which makes it useful for applications where different subsets of items are administered to different groups of respondents. The model also need to provide an accurate goodness of fit to the observed responses, and here too there may be an abundance of choice. For the Rasch model, tests of goodness of fit have been proposed that including Andersen’s (1973) likelihood ratio test (Glas & Verhelst, 1995), nonparametric tests (Ponocny, 2001; Verhelst et al., 2007), tests for specific model violations (Glas, 1988; Van den Wollenberg, 1979), and tests specifically designed to deal with sparse observations (Maydeu-Olivares & Joe, 2005, see Debelak, 2019; Suáres-Falcón & Glas, 2003 for an overview). Each of these tests assesses different dependencies in the observed response distributions and may be sensitive to different model violations. For example, Glas (1988) proposed a statistics, specifically designed to target the assumption of *local independence* (LI) by utilizing the information contained in the conditional bivariate distributions of pairs of items, given each sum score. Although found to be powerful in detecting violations of the Rasch model assumptions, for larger numbers of items the statistic is computationally demanding and the observations to which the statistic pertains become more sparse, limiting the asymptotic properties of the test statistic.

A similar problem occurs in factor analysis, where the estimation of the expected frequencies of the discrete responses involves high-dimensional (numerical) integration which becomes cumbersome for more items. Jöreskog and Moustaki (2001) and Katsikatsou et al. (2012) proposed a test statistics based only on the second-order moment to overcome these difficulties, but this procedure is also associated with loss of power for detecting model violations. These examples illustrate some of the tradeoff involved in the goodness-of-fit assessment when analyzing response data.

In this paper, the main focus is on Mokken’s (1971) model of *monotone homogeneity* (MH) for binary test data. In addition to the assumptions UD and LI, the model assumes latent *monotonicity* (M). The MH model is nonparametric in the sense that it does not require the response functions to belong to a particular parametric family. Further, the MH model is useful for applications that require ordinal inferences, as it implies a stochastic ordering on the latent variable by the sum score across the items (Ghurye & Wallace, 1959; Grayson, 1988; Huynh, 1994; Ünlü, 2008). The assumptions that constitute the MH model are shared by a wider range of models for response data, including the Rasch model and the three-parameter logistic model (Lord & Novick, 1968). These assumptions imply that all covariances between the test items are nonnegative. This testable property of the MH model for pairs of items is routinely used to assess the validity of the MH model assumption by means of inspecting the scalability coefficients (Loevinger, 1948; Mokken, 1971; Warrens, 2008) in Mokken scale analysis (Mokken & Lewis, 1982; Molenaar & Sijtsma, 2000; Sijtsma & Molenaar, 2002; Van der Ark, 2007). In Mokken scale analysis, any scalability coefficient that is below a predetermined lower bound (usually at 0.30) is flagged as a model violation that discredits the MH model, and any model that is a special case of the MH model (Junker & Sijtsma, 2001).

A problem with Mokken scale analysis based on the scalability coefficients is the somewhat arbitrary choice for the lower bounds of the coefficients. For example, Hemker et al. (1995) found that the default value of 0.30 does not always suffice to recover a unidimensional scale. Smits et al. (2012) also warn to be cautious about making inferences about the dimensionality of a test based on an automated evaluation of scalability coefficients. Tighter lower bounds for the scalability coefficients can be obtained from the requirement of *nonnegative partial correlations* (NPC; Ellis, 2014, 2015; Brusco et al., 2015). Like the scalability coefficients, the partial correlation is implied to be nonnegative under the MH model, but the property NPC takes into consideration the higher-order moments contained in the trivariate distributions of item triplets. As a consequence, a violation flagged by the property of NPC may remain undetected when only evaluating the covariances between item pairs.

Beside the scalability coefficients and NPC, other observable properties have been proposed that allow the assumptions of the MH model to be tested. For example, the property of *manifest monotonicity* (MM; Junker, 1993; Junker & Sijtsma, 2000) proposes that the regression of each of the item variables is a non-decreasing function of the sum of the remaining variables or *rest score*. Holland and Rosenbaum (1986) provide an overview of properties of multivariate positive dependence that are implied by the MH model, with *conditional association* (CA; Holland and Rosenbaum, 1986; Rosenbaum, 1984) being the most restrictive of these properties for binary response data. Below, we show that the observable property CA also implies MM and NPC (Ellis, 2015). Because the MH model cannot be directly evaluated, we rely on these observable properties to make inferences about the validity of the MH model assumptions (Sijtsma & Van der Ark, 2017). A testable latent class version of the MH model was proposed by Croon (1990, 1991); see also Hoijtink and Molenaar (1997) and Vermunt (2001), which requires a prior specification of the number of discrete latent classes. Global tests for some observable properties implied by the MH model have also been proposed. These global tests include both likelihood ratio tests for CA and MM (Bartolucci & Forcina, 2005; Tijmstra et al., 2013) and Bayes factors for MM (Tijmstra et al., 2015).

The next section starts with the introduction of the various observable properties that are implied by the MH model, and it will be shown how these properties are hierarchically related, with the property of CA imposing the tightest constants on the distribution of item responses. Because all the observable properties are implied by CA, each of these properties can be considered to be an incomplete test of CA (Maraun et al., 1998). Due to the number of restrictions imposed by CA and sparse observations associated with many of these restrictions, it is argued that the practical assessment of the MH model assumptions relies on incomplete tests for CA. In Sect. 2, we investigate the loss of information associated when, instead of CA, an incomplete test of CA is used, for which the complexity of the observable properties is defined as the agreement of the properties with a wider range of patterns of data. In Sect. 3, we look at the sensitivity of the various properties to violations of the MH model assumptions. The results of these studies are summarized and discussed in Sect. 4 along with their implications.

## Properties of Multivariate Dependence

In this section, seven distinct observable properties are defined for binary test data, all of which are implied by the MH model. Let $\varvec{X}=(X_1,\ldots ,X_J)$ be the random vector containing binary item response variables $X_i$. Also, let $\varvec{\Theta }$ denote the random vector of latent variables, with

$$\begin{aligned} p(\varvec{x})=P(\varvec{X}=\varvec{x})=\int P(\varvec{X}=\varvec{x}|\varvec{\Theta }=\varvec{\theta })\hbox {d}F(\varvec{\theta }). \end{aligned}$$

The assumption of LI states that the variables $X_1,\ldots ,X_J$ are locally or conditionally independent, given $\varvec{\Theta }=\varvec{\theta }$. Further, let $P(X_i=1|\varvec{\Theta }=\varvec{\theta })$ denote the *i*th response function, then the assumption M is satisfied whenever all *J* response functions are (element-wise) non-decreasing in $\varvec{\theta }$, and assumption UD holds if $\varvec{\Theta }=\Theta$ (i.e., scalar valued). The MH model is defined by the assumptions UD, LI, and M (Mokken, 1971).

It will be shown how the observable properties are related to each other, with property CA being the most restrictive of these properties. Next, several practical limitations will be discussed that relate to the number of inequality restrictions the properties impose on $\varvec{p}$ and the problem of sparseness of observation. Finally, to account for these practical limitations, the assessment of the trivariate distributions of all triplets of item is considered, adding two more distinct properties for assessing the MH model assumption.

### Observable Properties

Let $\varvec{p}$ be a vector, which has as its elements $p_k=p(\varvec{x})$, arranged in lexicographical order of $\varvec{x}$ (i.e., scores on the right run faster from zero to one). Then, $\varvec{p}$ contains the multinomial probabilities parameters for the distribution of the frequencies of $\varvec{X}=\varvec{x}$, with the restriction $\varvec{1}^\prime \varvec{p}=1$ (Holland, 1990). Each of the observable properties that are discussed below differs with respect to the additional restrictions they impose on $\varvec{p}$.

#### (Conditionally) Associated Random Variables

Esary et al. (1967) defined $\varvec{X}$ to be *associated* (A), if the covariance between any pair of binary non-decreasing functions of $\varvec{X}$ is nonnegative. A conditional version of property A was proposed by Holland and Rosenbaum (1986) and Rosenbaum (1984), where $\varvec{X}$ is said to be CA, if for any partition $\varvec{X}=(\varvec{Y},\varvec{Z})$, the variables $\varvec{Y}$ are associated, given any arbitrary function of $\varvec{Z}$.

Assume that $\varvec{p}>\varvec{0}$, then CA can be concisely expressed in terms restricted log-odds ratios, as

1 $$\begin{aligned} {\mathbf {K}}\ln ({\mathbf {M}}\varvec{p})\ge \varvec{0}, \end{aligned}$$

with ${\mathbf {K}}={\mathbf {I}}_v\otimes (1,-1,-1,1)$ (Kronecker product), ${\mathbf {I}}_v$ is the identity matrix of dimensions equal to the number of restrictions *v* imposed by CA, and ${\mathbf {M}}$ is a binary design matrix (Bartolucci & Forcina, 2005). Each of the consecutive four rows of the matrix ${\mathbf {M}}$ in (1) correspond to a particular restriction imposed on $\varvec{p}$ by property CA, with $v=(2^d-1)J(J-1)/2$ and $d=2^{J-2}$. For example, in case $J=2$, ${\mathbf {M}}={\mathbf {I}}_4$ and (1) yields $\ln p_1-\ln p_2-\ln p_3+\ln p_4\ge 0$.

Walkup (1968) characterized property A in terms of a collection of pairs of binary non-decreasing functions. For $J=3$, there are nine such pairs of functions. The constraints these functions impose correspond to restrictions on $\varvec{p}$ that can be expressed as (1), with the matrix ${\mathbf {M}}$ equal to

2 $$\begin{aligned} \left[ \begin{array}{c} (1,1)\otimes {\mathbf {I}}_4\\ {\mathbf {I}}_2\otimes (1,1)\otimes {\mathbf {I}}_2\\ {\mathbf {I}}_4\otimes (1,1)\\ {\mathbf {I}}_2\otimes ((1,0)^\prime \otimes (1, 1),{\mathbf {I}}_2)\\ {\mathbf {I}}_2\otimes ({\mathbf {I}}_2, (0, 1)^\prime \otimes (1, 1))\\ ({\mathbf {I}}_2\otimes (1, 0)^\prime \otimes (1, 1),{\mathbf {I}}_4)\\ ({\mathbf {I}}_4, {\mathbf {I}}_2\otimes (0, 1)^\prime \otimes (1, 1))\\ ((1, 1)\otimes (1, 0)^\prime \otimes {\mathbf {I}}_2,{\mathbf {I}}_4)\\ ({\mathbf {I}}_4,(1, 1)\otimes (0, 1)^\prime \otimes {\mathbf {I}}_2) \end{array}\right] . \end{aligned}$$

The last row in (2), for example, corresponds to the restriction

$$\begin{aligned} \ln p_1-\ln (p_3+p_5+p_7)-\ln p_2+\ln (p_4+p_6+p_8)\ge 0, \end{aligned}$$

or equivalently, Cov$(1-(1-X_1)(1-X_2),X_3)\ge 0$. For $J=4$, Walkup (1968, pp. 1400–1401) enumerated $v=99$ pairs of binary non-decreasing functions to characterize property A.

#### Multivariate Totally Positive

Next, consider the property of *multivariate totally positivity of order 2* (MTP$_2$; Karlin & Rinott, 1980) for a random vector $\varvec{U}$. The density $f(\varvec{u})$ is said to be $\hbox {MTP}_2$, if $f(\varvec{u})f(\varvec{v})\le f(\max (\varvec{u},\varvec{v}))f(\min (\varvec{u},\varvec{v}))$, for all outcomes $\varvec{u},\varvec{v}$, and with the minimum and maximum applied element-wise. For bivariate densities, the property is called $\hbox {TP}_2$ and corresponds to a *monotone likelihood ratio ordering* (MLR) in case the joint density is strictly positive (Karlin, 1968; Sarkar, 1969). This MLR property is relevant as it is the property used by Grayson (1988) to establish the stochastic ordering on $\Theta$ by the sum scores $S=X_1+\cdots +X_J$ under the MH model.

For the binary random vector $\varvec{X}$, assume that $\varvec{p}>\varvec{0}$. Then, (1) can also be used as an expression for $\hbox {MTP}_2$, by omitting the matrix ${\mathbf {W}}$ in the algorithm by Bartolucci and Forcina (2005, p. 41) for constructing matrix ${\mathbf {M}}$, and adjusting *v* accordingly. The $\hbox {MTP}_2$ property then corresponds to the requirement that Cov$(X_i,X_j|\varvec{Z}=\varvec{z})\ge 0$, for any partition $\varvec{X}=(X_i,X_j,\varvec{Z})$ and any vector $\varvec{z}$.

For a multidimensional vector $\varvec{\Theta }$, Holland and Rosenbaum (1986, Theorem 7) showed that the assumptions of LI and M imply that $\varvec{X}$ satisfies the property of $\hbox {MTP}_2$, if $\varvec{\Theta }$ is $\hbox {MTP}_2$. Also, $\varvec{X}$ is $\hbox {MTP}_2$, whenever $(\varvec{X},\varvec{\Theta })$ satisfying a particular higher-order factor structure (Ellis, 2015). These results imply that the property of $\hbox {MTP}_2$ is not confined to unidimensional models only.

#### Nonnegative Covariances

Equation (1) can also be used to restrict the bivariate distributions of pairs of item variables $X_i$ and $X_j$, such that Cov$(X_i,X_j)\ge 0$, for all $1\le i<j\le J$. Let

$$\begin{aligned} {\mathbf {T}}_{ij}=\bigotimes _{k=1}^J{\mathbf {T}}_{ijk}\text{, } \text{ with } {\mathbf {T}}_{ijk}=\left\{ \begin{array}{cl}{\mathbf {I}}_2 &{}\text{ if } \text{ either } i=k \text{ or } j=k\\ (1,1) &{}\text{ otherwise, }\end{array}\right. \end{aligned}$$

and let the matrix ${\mathbf {M}}$ be obtained by stacking on top of one another all matrices ${\mathbf {T}}_{ij}$. With this matrix ${\mathbf {M}}$ and $v=J(J-1)/2$, expression (1) imposes the restriction of the property of *nonnegative covariances* (NC), which implies that all the scalability coefficients are nonnegative (Mokken, 1971; Sijtsma & Molenaar, 2002).

#### Manifest Monotonicity

The observable property MM pertains to the regression of each $X_i$ on $S-X_i$, with $S=X_1+\cdots +X_J$. Junker (1993) showed that MM provides a partial characterization of a general class of latent variable models that include the MH model. To show CA implies MM, let $R=S-X_i-X_j$. Then, CA implies for all $R=r$, that

$$\begin{aligned} \begin{array}{l}P(X_i=0,X_j=0,R=r)P(X_i=1,X_j=1,R=r)\\ \quad \ge P(X_i=0,X_j=1,R=r)P(X_i=1,X_j=0,R=r),\end{array} \end{aligned}$$

or equivalently $P(X_i=1|S-X_i=r)\le P(X_i=1|S-X_i=r+1)$. The inequalities imposed by MM thus correspond to a selection of consecutive rows of ${\mathbf {M}}$ for CA. For example, for $J=3$, matrix ${\mathbf {M}}$ for MM becomes

3 $$\begin{aligned} \left[ \begin{array}{c} \varvec{I}_2\otimes (\varvec{I}_2,(1,0)\otimes (0,1)^\prime )\\ \varvec{I}_2\otimes ((0,1)\otimes (1,0)^\prime ,\varvec{I}_2)\\ (\varvec{I}_4,\varvec{I}_2\otimes (1,0)\otimes (0,1)^\prime )\\ (\varvec{I}_2\otimes (0,1)\otimes (1,0)^\prime ,\varvec{I}_4)\\ (\varvec{I}_4,(1,0)\otimes (0,1)^\prime \otimes \varvec{I}_2)\\ ((1,0)\otimes (0,1)^\prime \otimes \varvec{I}_2,\varvec{I}_4) \end{array}\right] . \end{aligned}$$

Unlike the other observable properties that have been discussed thus far, MM for all test item does not imply that MM also holds for any subset of item. For example, for $J\ge 3$, MM does not imply NC nor the other way around.

#### Strongly Positive Orthant Dependency

Holland (1981) proposed a generalization of the MH model, by relaxing the LI condition. His approach to modeling the dependencies between the item variables uses clusters of item variables with outcomes of all zeros or ones. Let $\varvec{V}$ contain a selection of variables from $\varvec{X}$ and consider the partition $\varvec{V}=(\varvec{Y},\varvec{Z})$. Besides UD, also assume that both

4a $$\begin{aligned}&P(\varvec{V}=\varvec{1}|\Theta =\theta ) \text{ is } \text{ non-decreasing } \text{ in } \theta \text{, } \text{ and } \end{aligned}$$

4b $$\begin{aligned}&P(\varvec{V}=\varvec{0}|\Theta =\theta ) \text{ is } \text{ non-increasing } \text{ in } \theta , \end{aligned}$$

for any selection $\varvec{V}$. Then, Holland (1981) showed that these assumptions together with the assumption of *local nonnegative dependence* (LND) coincide with following three inequalities:

5a $$\begin{aligned}&P(\varvec{V}=\varvec{1})\ge P(\varvec{Y}=\varvec{1})P(\varvec{Z}=\varvec{1}), \end{aligned}$$

5b $$\begin{aligned}&P(\varvec{V}=\varvec{0})\ge P(\varvec{Y}=\varvec{0})P(\varvec{Z}=\varvec{0})\text{, } \text{ and } \end{aligned}$$

5c $$\begin{aligned}&P(\varvec{Y}=\varvec{1},\varvec{Z}=\varvec{0})\le P(\varvec{Y}=\varvec{1})P(\varvec{Z}=\varvec{0}), \end{aligned}$$

for any partition of the selected variables $\varvec{V}=(\varvec{Y},\varvec{Z})$, where the assumption LND is obtained from (5a–5c) by conditioning each term on $\Theta =\theta$.

The observable property defined by (5a–5c), for any $\varvec{V}=(\varvec{Y},\varvec{Z})$ implies *strongly positive orthant dependency* (SPOD; Joag-Dev, 1983), with the latter obtained by taking $\varvec{V}=\varvec{X}$ (Block & Fang, 1990). Following Holland and Rosenbaum (1986, p. 1531), we refer to the property defined by (5a–5c) as SPOD, but have it understood that it applies to any subset of item variables from $\varvec{X}$.

The property SPOD can be expressed in terms of the log-odds ratios in (1) by appropriately adjusting matrix ${\mathbf {M}}$ and *v*. For example, for $\varvec{V}=(X_i,X_j)$, all three inequalities coincide with Cov$(X_i,X_j)\ge 0$. For $J=3$, let $\varvec{Y}=X_1$ and $\varvec{Z}=(X_2,X_3)$, so that (5a) and (5b) imply that

$$\begin{aligned} \begin{array}{l}\ln p_8-\ln p_4-\ln (p_5+p_6+p_7)+\ln (p_1+p_2+p_3)\ge 0 \text{ and }\\ \ln p_1-\ln p_5-\ln (p_2+p_3+p_4)+\ln (p_6+p_7+p_8)\ge 0,\end{array} \end{aligned}$$

respectively. These two inequalities hold, if and only if (5c) holds, for $\varvec{Y}=(X_2,X_3)$ and $\varvec{Z}=X_1$, and $\varvec{Y}=X_1$ and $\varvec{Z}=(X_2,X_3)$, respectively. Hence, for $J=3$, SPOD reduces to inequality (5c), for all $\varvec{V}=(\varvec{Y},\varvec{Z})$.

#### Nonnegative Partial Correlations

Unlike the observable properties discussed above, NPC does not lend itself to be expressed as restrictions on the log-odds ratios. Instead, consider the selection of variables $(X_i,X_j,X_k)$ from $\varvec{X}$. Then, for any such selection of variables, the property NPC requires that

6 $$\begin{aligned} \text{ Cov }(X_i,X_j)\text{ Var }(X_k) \ge \text{ Cov }(X_i,X_k)\text{ Cov }(X_j,X_k), \end{aligned}$$

which each selected variable taking on the role of $X_k$ once (Ellis, 2014). NPC holds, whenever all trivariate distributions of triplets of response variables satisfy $\hbox {MTP}_2$ (Ellis, 2015).

### Relationships Between the Observable Properties

**Fig. 1:**
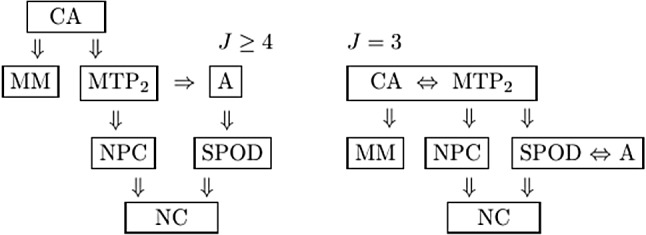
Hierarchical relationships between the observable properties, for *J* binary variables.

All observable properties for the binary response data above are implied by CA (Holland & Rosenbaum, 1986, p. 1536). Figure 1 (left) shows an overview of the observable properties and their relationships, for $J\ge 4$. The property MM is implied by CA, but MM neither implies, nor is implied by any of the other properties. In Fig. 1, NPC pertains to the trivariate distributions of all triplets of items, and NC pertains to the bivariate distributions of all pairs of items. The remaining observable properties apply to the multivariate distribution of all the *J* item variables. In case $J=2$, all the properties coincide with Cov$(X_1,X_2)\ge 0$. For $J=3$, binary random variables, Ellis (2015) showed that the properties CA and $\hbox {MTP}_2$ coincide. Also, the properties A and SPOD coincide (‘Appendix’), as shown in Fig. 1 (right).

### Practical Considerations

**Fig. 2:**
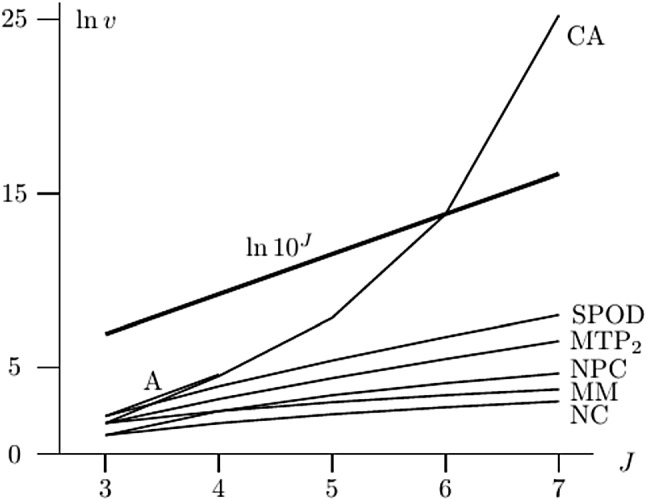
The number of restrictions imposed by the observable properties as a function of *J*.

Figure 2 also shows the natural logarithm of the number of restrictions *v* imposed on the multivariate distribution of the item variables by the observable properties in Fig. 1. The bold line is included for reference and shows that the number of restrictions imposed by CA fast exceeds $10^J$ for $J>6$. This means that an exhaustive or complete test of CA is practically infeasible for more than five items (Bartolucci & Forcina, 2005; De Gooijer & Yuan, 2011).

The many inequality restrictions imposed by the various properties limit the use of likelihood ratio tests (Bartolucci & Forcina, 2000, 2005; Tijmstra et al., 2013) that require the estimation of $\varvec{p}$ under all constraints imposed by the restriction. Also, obtaining the distribution of the test statistics often involves simulations, where the problem is similar to Bayesian methods for testing the properties (e.g., Tijmstra et al., 2015, for MM), in that the agreement to all *v* restrictions need to be assessed for many samples of $\varvec{p}$. For local (diagnostic) tests, as performed in Mokken scale analysis (Molenaar & Sijtsma, 2000; Van der Ark, 2007), the problem induced by the many restrictions is that of multiple testing (Ellis, 2014).

Beside the many restrictions, another problem for assessing the observable properties relates to sparseness of observations. Because the number of response patterns $\varvec{x}$ increases exponentially with the number of items, many of these response patterns will be expected to have sparse observations, even for large sample sizes. The sparse observations may thus not only limit the extent to which one can rely on the asymptotic results of a likelihood ratio test, but also make the results of locally performed tests sensitive to sampling error.

**Fig. 3:**
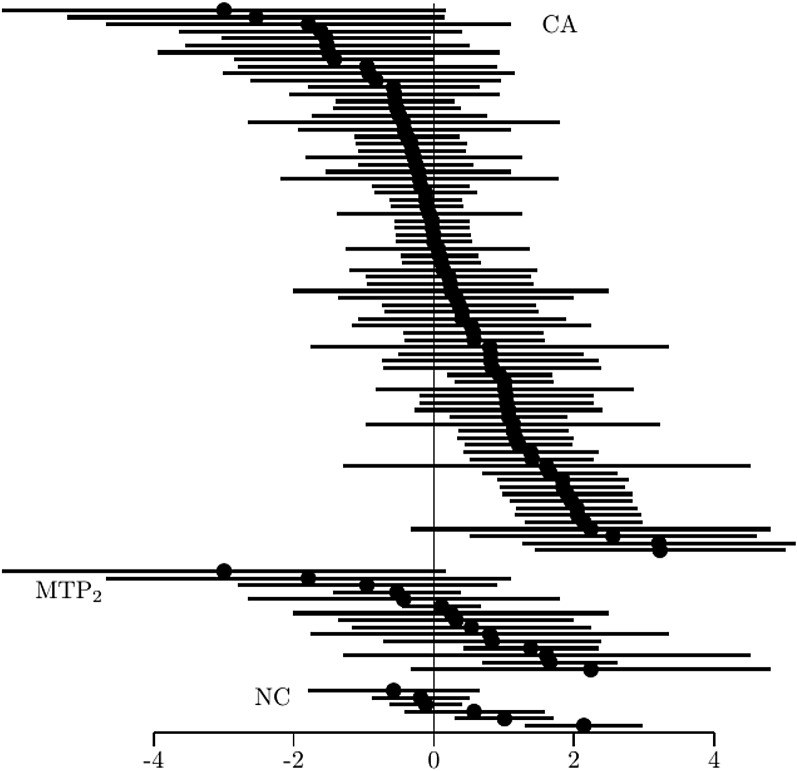
Log-odds ratios for the properties CA, $\hbox {MTP}_2$, and NC (for each in ascending order), along with the 95% confidence intervals.

Not all properties are equally sensitive to sparse observation. By pertaining only to the (marginal) bivariate distributions, the assessment of property of NC will generally involve fewer number of sparse observations than $\hbox {MTP}_2$, for example, where each restriction involves the joint distribution of four response patterns. For illustration, data on the performance of 425 pupils on four transitive reasoning tasks (Length) were analyzed (Verweij et al., 1996, available from the mokken package, Van der Ark, 2007). Two of the vectors $\varvec{x}$ contained no observations, so that the active number of restrictions of CA was reduced by 12–78. Figure 3 shows the 78 estimated log-odds ratios in ascending order, along with their 95% confidence interval. The figure shows that there are 33 violations of CA; one significant violation. Figure 3 also shows the 15 out of 24 (active) logs-odds ratios for property $\hbox {MTP}_2$ and the six estimated values for NC. Comparing the results of $\hbox {MTP}_2$ to NC clearly illustrates how the property NC is more robust to sampling error, as reflected by the narrow confidence intervals compared to those for $\hbox {MTP}_2$. However, NC is also associated with a substantial loss of power, with the log-odds ratios generally located more to the right.

### Properties for Trivariate Distributions of Item Triplets

The previous section showed that, on the one hand, the property NC overcomes the problem of sparse observations by pertaining to the bivariate (marginal) distributions of pairs of items, but is also associated with a substantial loss of information about the validity of the MH model assumptions. On the other hand, the property $\hbox {MTP}_2$ does appear to be more powerful in detecting violations of the model assumptions, but is rather sensitive to sparseness of observations, rendering it sensitive to sampling error.

The property NPC utilizes the information contained in the trivariate distributions of all triplets of item variables and thereby strikes a balance between the practical limitations that affect property NC and $\hbox {MTP}_2$. Property NPC imposes tighter constraints on $\varvec{p}$ than NC and might therefor provide a more powerful test for detecting violations of the MH model assumptions. Also, the trivariate distribution of item triplets will generally contain few sparse observations for sufficiently large sample sizes, $N>200$, say.

**Fig. 4:**
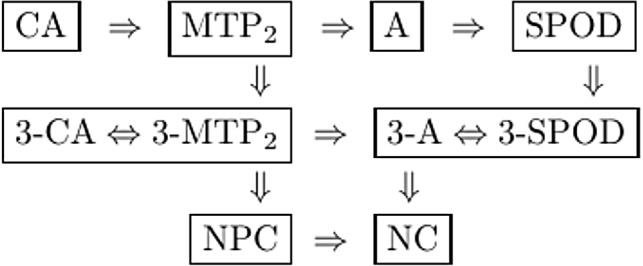
Hierarchical relationships between the observable properties (excluding MM), for $J\ge 4$ binary variables.

Like property NPC, consider applying the multivariate observable properties to the trivariate distributions of all triplets of item variables, and let 3-CA denote the property CA applied to the trivariate distributions of all triplets of items (similar for the other properties). Then, the properties applied to the trivariate distributions are related as shown in Fig. 4. The top two rows in Fig. 4 coincide in case $J=3$.

## Incomplete Tests of Conditional Association

In this section, the tightness of the constraints imposed on $\varvec{p}$ by the observable properties is investigated. With property CA implying all the other properties considered in the previous section, the other properties for assessing the MH model assumptions can be considered to be incomplete tests of CA; in the sense the properties can be obtained by relaxing some of the restrictions imposed by CA (Maraun et al., 1998). In practice, we rely on such incomplete tests, due to the large number of restrictions CA imposes. However, the number of inequality restrictions does not provide a clear indication of the tightness of the constraints imposed by the observable properties. For example, for $J=4$ property A imposed 99 restrictions, which are all implied by the 24 constraints imposed by $\hbox {MTP}_2$. Figure 1 shows the hierarchical relationships of the observable properties, but it does not show how much information is lost when, instead of CA, an incomplete test of CA is used to make inferences about the MH model assumptions. The advantage of the use of incomplete tests is that their assessment generally involves fewer inequality restrictions, and these incomplete tests are generally less sensitive to sparse observations. As a consequence, incomplete tests of CA are practically useful, but only to the extent that they are not associated with a substantial loss of information about CA. Such a loss of information would namely result in loss of power when assessing the MH model assumptions.

In the application of their likelihood ratio procedure, Bartolucci and Forcina (2005) observed that only a few CA restrictions were ‘activated’ in addition the restrictions imposed by $\hbox {MTP}_2$. This suggests that little information may be lost when $\hbox {MTP}_2$ is assessed, instead of property CA. Here, the tightness of the constraints imposed on $\varvec{p}$ is investigated in terms of model complexities, which provides a general assessment of the observable properties that does not rely on the data. With the observable properties all impose inequality restrictions on the probabilities associated with the multinomial frequencies, we can think of each of these properties as a model for the multinomial response frequencies and rephrase the choice for an incomplete test for the MH model assumptions as a model selection problem.

In general, model selection involves a tradeoff between the *goodness of fit* of the models under consideration and the model *complexities*. A model is selected, if it can accurately predict future data. This requires accurate model-data fit, while also providing a description of the data that is as simple as possible (Occam’s razor), as not to overfit the data. Statistics that balance goodness of fit against model complexity include Akaike’s (1974) AIC and Schwarz’s (1978) BIC, where the goodness of fit is expressed by the likelihood function, and the model is penalized by the estimated number of parameters. Complexity, however, involves more than the number of estimated parameter (Myung et al., 2005). For example, Bonifay and Cai (2017) found that different parametric models for response data that had the same number of parameters differed in the extent to which they fit diverse patterns of data. They thereby showed that model complexity is only partly described by the number of model parameters (Pitt et al., 2002; Preacher, 2006). Similar to the idea of *fitting propensity* suggested by Preacher (2006), we here define the complexities of the observable properties as the proportion of samples from the (unconstrained) multinomial model that satisfy the inequality constraints of the observable properties. By assigning a distribution to the multinomial probability parameters, this notion of complexity corresponds to the definition of model complexity for Bayes factors, with the distribution of the multinomial parameters taking up the role of the encompassing prior (Hoijtink, 2011; Klugkist & Hoijtink, 2007). A more complex property is then said to impose looser constraints on the outcomes, thus fitting a wider range of patters of data. In this respect, a higher complexity means that the property is generally less sensitive to model violations. Hence, property CA is the least complex of the properties considered, and NC is the most complex.

### On the Complexity of the Observable Properties

A simulation study was performed as an initial assessment of the complexities of the observable properties, for $J=3$. A total of one million vectors $\varvec{p}$ where samples from a flat Dirichlet distribution, with $\varvec{p}>\varvec{0}$ and $\varvec{1}^\prime \varvec{p}=1$. These samples provided a uniform coverage of the outcome space of $\varvec{p}$ (cf. Bonifay & Cai, 2017). Subsequently, for each vector $\varvec{p}$, all the observable properties in Fig. 1 (right) were assessed. The proportion of samples that satisfy a given observable property then provides an indication of the complexity of the property.

**Fig. 5:**
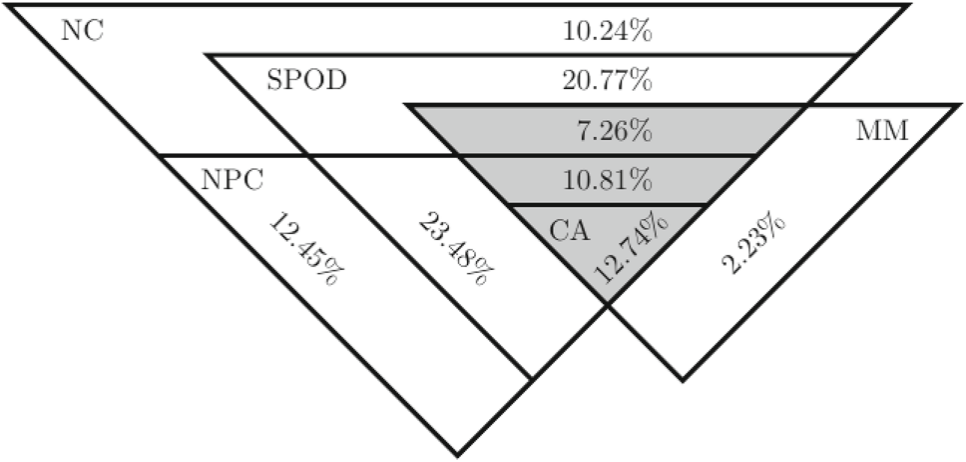
Triangular Venn diagram of properties in Fig. 1 ($J=3$), with the overlap between NC and MM in gray, with the conditional percentages, given either NC or MM (or both).

The results of the simulation show that a total of 163,627 samples (16.36%) satisfy either NC or MM or both, with a small percentage (0.36%) that only satisfied MM, and about 5.04% that satisfy both NC and MM. Figure 5 shows the overlap between the observable properties, with the conditional percentages, given that either NC or MM or both are satisfied. Note that the intersection of NC and MM is contained in SPOD. In ‘Appendix,’ it is proven that this is always the case. Figure 5 shows that SPOD accounts for about 75.08% of all samples that satisfy either NC or MM. Of the 10 million samples (unconditionally), CA was satisfied by about 2.09% of the samples. The constraints imposed by CA are considerably tighter than those imposed by the other observable properties, with no one property containing more than 40% (38.56% for MM) of samples that also agree with CA. If both NC and MM satisfied, then about 41.35% of these samples also satisfy CA.

### Scalability Coefficient

Rather than using an incomplete test of CA to assess the MH model assumptions, the associations between the response variables can be expressed by a statistics, like a scalability coefficient. A desirable property of such a statistic would be that it is related to the tightness of the imposed bounds on $\varvec{p}$ (Kimeldorf & Sampson, 1989), such that the value of the statistic corresponds to the hierarchical relationship in Fig. 1. To assess whether property CA can be reliably inferred from the value of scalability coefficients *H*, the coefficient was computed for each of the previously sampled vectors $\varvec{p}$ (e.g., Roskam et al., 1986, p. 266).

**Fig. 6:**
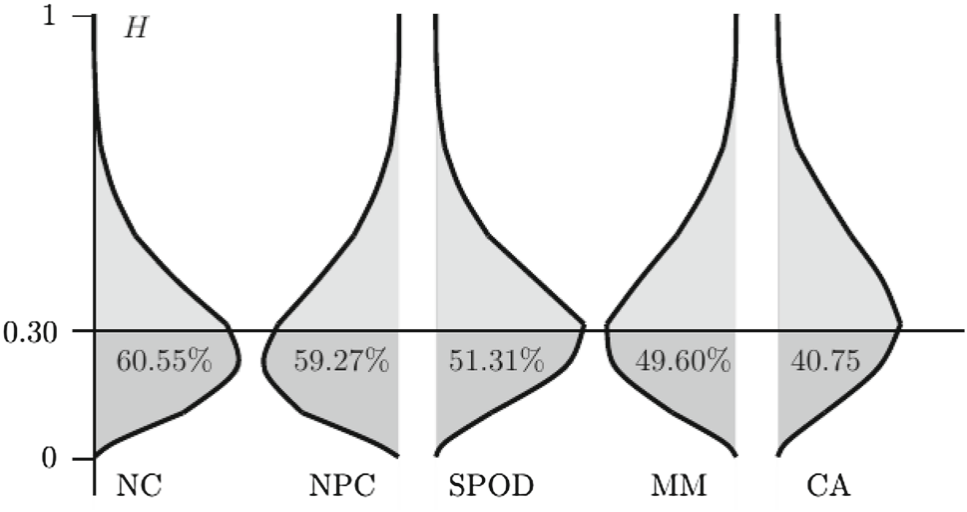
Conditional densities (vertically displayed) of the scalability *H*, given the properties in Fig. 1 ($J=3$), along with the percentages $H<0.30$.

Figure 6 shows the estimated conditional densities of *H*, given each of the observable properties in Fig. 1 (right). Although the ordering of these densities roughly agrees with the hierarchical relationships between the properties, Fig. 6 shows that the densities have a considerable overlap. This means that it is practically impossible to reliably infer which property holds, given the value of *H*. Moreover, the value of coefficient *H* was below the default recommended value of 0.30 for 40.75% of the cases for which property CA was satisfied.

### Manifest Monotonicity

Property MM was found to be the least complex of the incomplete tests of CA for $J=3$, imposing the tightest constraints on $\varvec{p}$ after CA. Here, we further explore the discrepancy in complexity between MM and CA as *J* increases. To this end, a Gibbs sampler was employed to sample 10,000 vectors $\varvec{p}$ under the constraints imposed by MM and assess the percentage of these samples that also satisfy CA. We first explain the Gibbs sampling procedure (cf. Ligtvoet & Vermunt, 2012; Hoijtink & Molenaar, 1997).

*Gibbs sampler* Suppose we wish to sample a vector $\varvec{p}$ from a flat Dirichlet distribution under the constraints imposed by *v* inequality restrictions. Also, suppose we already have the vector $\varvec{q}$ that satisfies these constraints. Then, we can sequentially sample the values $p_j$ by following the next three steps. First, compute from the inequalities imposed on $p_j$ the maximum lower bound $a_j$ and the minimum upper bounds $b_j$, using the values $q_1,\ldots ,q_{j-1},q_{j+1},\ldots , q_{2^J}$. For example, for $J=3$, the element $p_2$ is bounded from above by MM by the first restriction in (3): $p_2\le q_1(q_7+q_7)/q_5-q_3$. Second, sample a value $q^*_j$ from a gamma distribution (unit shape) that is truncated from below by $\max (0,a_j)$ and from above by $b_j$. From this, the new vector $\varvec{q}=\varvec{q}^*/\varvec{1}^\prime \varvec{q}^*$ is obtained, with $\varvec{q}^*=(q_1,\ldots ,q_{j-1},q_j^*,q_{j+1},\ldots , q_{2^J})^\prime$. Third, we have for $\varvec{p}$ the vector $\varvec{q}$ obtained by repeating the first two steps for all $p_j$.

To obtain the initial vector $\varvec{q}$ for the Gibbs sampler, a single sample is taken from the flat Dirichlet distribution, for which we assess the required restrictions. Those restrictions that are satisfied are then ‘activated’ and the Gibbs sampler is run using the active restrictions only, resulting in a new vector for which at least the active restrictions are satisfied. The vector $\varvec{q}$ is then obtained by repeating the Gibbs sampler and activating those (additional) restrictions that are satisfied at each step, until all *v* restrictions are active.

Recall that for $J=3$, 38.56% of the samples that satisfied property MM also satisfied CA. Of the 10,000 samples obtained from the Gibbs sampler for $J=4$, about 0.06% were found to also satisfy CA. Increasing the number of items to five further reduced this percentage to below 0.01%. The results strongly suggest that the discrepancy in complexity between the properties MM and CA increases as the number of items increases.

### The Distributions of Subsets of Item Variables

**Fig. 7:**
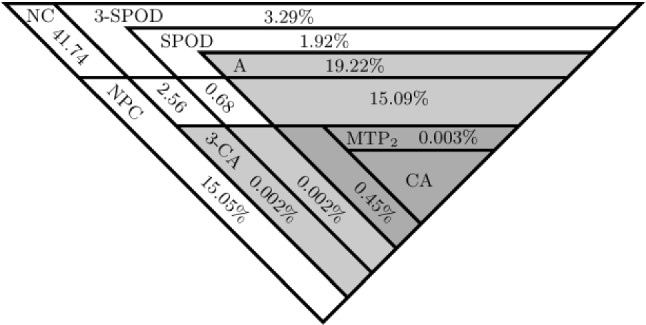
Triangular Venn diagram of properties in Fig. 4, with the conditional percentages, given NC. The properties A and 3-CA and their overlap are shown in gray.

The complexities of the properties are further investigated for $J=4$, which extends the results in Fig. 5 (excluding MM) and includes the properties $\hbox {MTP}_2$ and A, along with 3-CA and 3-SPOD for the trivariate distributions of all four triplets of item variables. A total of 10 million samples of the vector $\varvec{p}$ were obtained from a flat Dirichlet distribution. Of these 10 million samples, 343,556 (3.44%) satisfied NC. For these 343,556 samples, Fig. 7 shows the percentages of overlap between the observable properties. For example, the gray areas in Fig. 7 correspond to the properties A and 3-CA, where A accounts for about 34.76% of the samples that satisfy NC and the property 3-CA accounts for about 0.45%, with the latter, thus imposing considerably tighter constraints on $\varvec{p}$ (less complex). Of the samples that satisfy NC, both $\hbox {MTP}_2$ and CA were satisfied by less than 0.01%. After CA and $\hbox {MTP}_2$, the properties 3-CA and NPC imposed the tightest constraints on $\varvec{p}$, which were satisfied by, respectively, 0.45% and 33.83% of all samples that satisfied NC (0.02% and 1.16% of all 10 million samples). However, even for those samples that satisfied 3-CA, only about 0.77% also satisfied CA. Hence, for $J=4$, the results show that there exists a considerable gap between the complexity of property CA and any of the incomplete tests for CA (except $\hbox {MTP}_2$).

For $J>3$, the property CA implies $\hbox {MTP}_2$, but not the other way around. However, because of the small number of cases that satisfied $\hbox {MTP}_2$, none of the samples contained cases for which $\hbox {MTP}_2$ was satisfied and CA was not. To further investigate the distinction between the complexity of $\hbox {MTP}_2$ and CA, the Gibbs sampler (Sect. 2.3) was employed to sample 10,000 vectors $\varvec{p}$ under the constraints imposed by $\hbox {MTP}_2$. For $J=4$, the percentage of samples that satisfied CA was about 98.38%. Using the same procedure for $J=5$, this percentage slightly reduced to 94.48%, with the log-odds ratio of the largest observed violation of CA corresponding to a small effect size (Haddock et al., 1998; Hasselblad & Hedges, 1995). This result agrees with the observation mentioned earlier by Bartolucci and Forcina (2005).

## Sensitivity to Model Violations

All the observable properties considered in the previous section are implied by the MH model for binary response variables, such that the violation of any of these properties discredits the assumptions that define the MH model. The different properties may, however, not be equally sensitive to different model violations. Insights into the sensitivity of the observable properties to various model violations may aid the development of goodness-of-fit statistics for specific model assumptions.

### Violations of Local Independence

The MH model consists of the assumptions of LI, UD, and M. Holland (1981) suggested an alternative set of assumptions, consisting of LND, UD, and the monotonicity assumption of perfect scores in (4a) and (4b), which imply M. Here, LND relaxes the LI assumption, whereby LI is obtained from the LND assumption by replacing the inequality restrictions of LND by equalities (Holland 1981, Theorem 1). The alternative set of assumptions coincide with the observable property of SPOD, which means that SPOD corresponds to a model for which LI is not assumed to hold. Furthermore, the MH model implies CA, which in turn implies SPOD Rosenbaum (1984).

As was shown in the previous section, CA occupies only a very small section of the outcomes space that satisfies SPOD. Hence, CA is a priori unlikely to hold, given that the data satisfy a model that does not imply LI. Consequently, we may conclude that CA is sensitive to violations of the LI assumptions. Based on the results in Fig. 7 ($J=4$), the same may be concluded for $\hbox {MTP}_2$ and (tentatively) for 3-CA, as these properties show little overlap with SPOD.

Neither of the properties NC, NPC nor MM imply SPOD, which means that these properties may or may not hold, irrespective of SPOD. The properties may then be sensitive to violations of LI when modeled in a specific way, but not to violations of LI in general. Property MM, however, is shown in Fig. 5 to be almost completely encompassed by SPOD and thus may be found to be sensitive to violations of LI more generally. For Mokken scale analysis based on these properties, this means that a violation of NC or NPC discredits the MH model, but from this it cannot be concluded that the observed violation was due to a violation of the LI assumption.

### Violations of Unidimensionality

Holland and Rosenbaum (1986) referred to a model that satisfies LI and M, but allows $\varvec{\Theta }$ to be multidimensional, as a *monotone latent variable model*. They showed that any monotone latent variable model implies property $\hbox {MTP}_2$, if the density of $\varvec{\Theta }$ is $\hbox {MTP}_2$. A similar result was obtained by Ellis (2015), in case $(\varvec{X},\varvec{\Theta })$ satisfies a particular higher-order factor structure. This means that one cannot make inferences about the dimensionality of (the unobserved) $\varvec{\Theta }$ based on the confirmation of $\hbox {MTP}_2$ or any property it implies. Because of the minor discrepancy found between the properties $\hbox {MTP}_2$ and CA, the assessment of the dimensionality of $\varvec{\Theta }$ poses a real challenge for future research.

Another difficulty, when studying the influence of violations of UD, is that the addition of more latent variables in a model generally coincides with a violation of the LI assumptions when fitting a unidimensional model.

### Violations of Monotonicity

A small simulation study is performed to investigate the sensitivity of the observable properties to violations of assumption M. Given the assumptions of LI and UD, a choice needs to be made for the number of items, the distribution of the latent variable, and a way of inducing and quantifying violations of M. The results of the analysis on the sensitivity of the observable properties to violations of M highly depend on these choices. In order to make the results fairly generalizable across a wide range of choices of model specifications, a latent class approach is used (e.g., Croon, 1990; Heinen, 1993; Lazarsfeld, 1950). The approach consists of assuming a discrete distribution for the latent variable. By taking the number of latent classes to equal to the number of distinct response patterns, this approach is highly flexible with respect to the shape of the distribution of the latent variable and the shape of the response functions.

The choice for the number of items is motivated by the results on the complexities of the properties, which were shown to be very restrictive, especially for large numbers of items. By initially taking $J=4$, we may expect the latent class model to generate sufficient samples of the vector $\varvec{p}$ for which the properties hold, in order to compare the size of the violations of M between those cases where the property is violated to those cases where the property holds. For $J=3$, the results are similar to the ones presented here.

#### Procedure

**Fig. 8:**
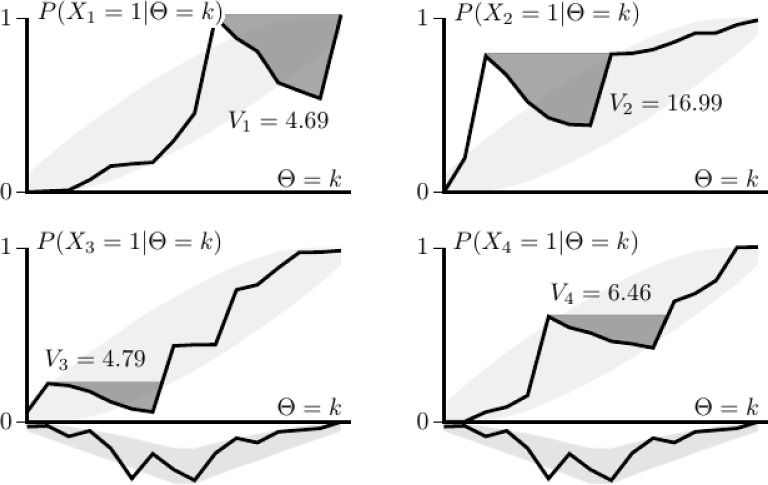
Example of four item response functions that violate M, with the density of $\Theta$ given below. The light-gray areas show the 95% intervals under which the functions were generated before inducing a violation of M. The dark-gray areas (above the local decreases) show the size of the violations of M, with $V_i$ expressing the size of the area weighted by the density of the latent variable.

For the distribution of $\Theta$, a vector $\varvec{c}=(c_1,\ldots ,c_{16})^\prime$ was sampled from a Dirichlet distribution, which contains the latent class proportions $c_k=P(\Theta =k)$. The parameters of the Dirichlet distribution were chosen, such that the middle latent classes had generally more support. Further, let $\varvec{b}_i=(b_{i1},\ldots ,b_{i16})^\prime$, with $b_{ik}=P(X_i=1|\Theta =k)$ sampled from a beta distribution, and with the elements in $\varvec{b}_i$ arranged in increasing order in agreement with assumption of M. Figure 8 shows an example of four response functions $P(X_i=1|\Theta =k)$, with in light gray the 95% intervals of the response functions under the simulation conditions, along with the intervals for the latent classes. To induce a violation of M, six adjacent element of $\varvec{b}_i$ were randomly selected, and reversely ordered, leading to locally decreasing response functions. Assuming LI, we then get $\varvec{p}={\mathbf {A}}\varvec{c}$, with ${\mathbf {A}}=(\varvec{a}_1,\ldots ,\varvec{a}_{16})$ and $\varvec{a}_k=(1-b_{1k},b_{1k})^\prime \otimes \cdots \otimes (1-b_{4k},b_{4k})^\prime$. A total of 10,000 such vectors $\varvec{p}$ were generated, each containing the multinomial parameters for the outcomes of the four item variables, with each response functions violating the assumption M.

To quantify the size of the violation of M, let $\varvec{d}_i=(b_{i1},d_{i2},\ldots ,d_{i16})^\prime$, with the values of $d_{i2},\ldots ,d_{i16}$ obtained sequentially as $d_{ik}=\max (d_{i,k-1},b_{ik})$. Then,

7 $$\begin{aligned} V_i=\varvec{c}^\prime (\varvec{d}_i-\varvec{b}_i)\times 100\%, \end{aligned}$$

which expresses the average probability (as percentage) required to compensate for the local decreases of the initial response function. Figure 8 shows for each item the value $V_i$, corresponding to the dark-gray area above the local decrease, weighted by the probability mass function of $\Theta$. For example, for the first two items in Fig. 8, $V_1=4.69$ and $V_2=16.99$, where the second response function shows a decrease at a denser region of $\Theta$.

#### Results

Let $V_{\text{ M }}$ denote the average value of $V_i$, across the four items. The results of the simulation show that ${\overline{V}}_{\text{ M }}=8.169$ across the 10,000 generated cases (with the 1st and 3th quartile at 6.389 and 9.700, respectively), which is about equal to the value of $V_{\text{ M }}$ obtained for Fig. 8.

Assessing the validity of the observable properties and evaluating the distributions of $V_{\text{ M }}$ for those cases for which the properties held true showed that the distributions of $V_{\text{ M }}$ were about the same for the properties 3-SPOD, A, and SPOD, and about the same for both $\hbox {MTP}_2$ and CA. The results of the simulation are therefore discussed further only for the properties NC, 3-CA, NPC, SPOD, MM, and CA.

**Fig. 9:**
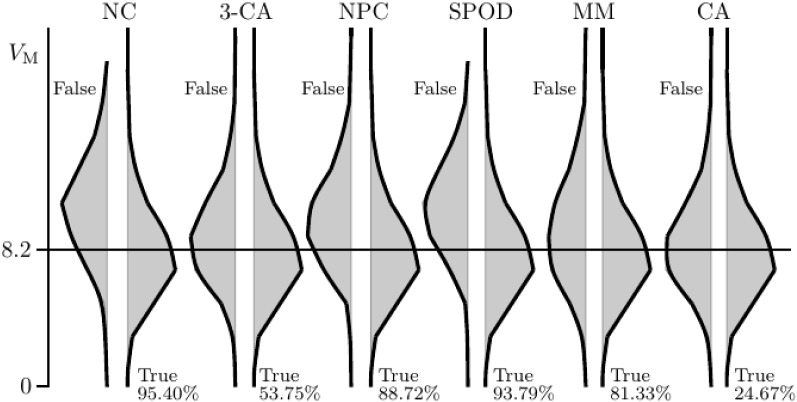
Conditions distributions of the size of the violations of M ($V_{\text{ M }}$), given that the properties NC, 3-CA, NPC, SPOD, MM, and CA hold (True; with percentage of cases) or are violated (False). Results for the properties 3-SPOD and A are similar as for 3-CA, and the results for $\hbox {MTP}_2$ are similar as for CA.

For each property, Fig. 9 shows the estimated densities of $V_{\text{ M }}$ (vertically displayed) in case the property was satisfied (True; false discovery) and in case it was violated (False). Figure 9 also shows the percentage of times each property was satisfied, with property CA satisfied about 24.67% of the time, 3-CA satisfied about half the time, and the remaining properties satisfied most of the time. The percentages listed in Fig. 9 roughly agree with the hierarchical ordering of the property in Fig. 1.

The differences of the violations $V_{\text{ M }}$ between the True cases and the False cases were found to be of a small to medium size for the properties 3-CA, MM, and CA, in in accordance to Cohen’s (1988) *d*. Figure 9 shows that the properties NPC, NC, and SPOD are most sensitive to the size of the violations of M, each corresponding to a large effect size, with the larges value $d=1.127$. Hence, SPOD is not sensitive to violations of M in a strict sense (only rejected about 6.21% of the time), but the property is more likely to be rejected when the violations of M are larger. This in contrast to property CA, which is generally more likely to be rejected, irrespective of the size of the violations. For practical purposes, however, it may be argued that a relative small violation of M should not matter. This would mean that CA may impose constraints on the observable data distribution that just are too restrictive. For example, one might only be interested in testing the MH model assumptions, because this model implies a MLR ordering on the latent variable by the sum score. Then, for the practical use of the sum score, the size of the violation of assumption M matters only to the extent to which it jeopardizes the MLR property.

#### The Monotone Likelihood Ratio Property

To assess the influence of the M assumption on the MLR property, the response functions that violate M are combined for each of the 10,000 cases to give an expression for the violation of property MLR, similar to $V_{\text{ M }}$. To this end, let ${\mathbf {E}}=\mathbf {HA}$, with element $e_{sk}=P(S=s-1|\Theta =k)$. Here, ${\mathbf {A}}$ is obtained from the simulation and ${\mathbf {H}}$ is a matrix to relate the vectors $\varvec{x}$ to their sum scores. Specifically, let ${\mathbf {H}}_1={\mathbf {I}}_2$ and ${\mathbf {H}}_{i+1}=[({\mathbf {H}}_i,\varvec{0}^\prime )^\prime ,(\varvec{0}^\prime ,{\mathbf {H}}_i)^\prime ]$, from which ${\mathbf {H}}={\mathbf {H}}_J$ is obtained sequentially. Then, vector $\varvec{b}_s=(b_{s1},\ldots ,b_{s16})^\prime$, with

$$\begin{aligned} b_{sk}=e_{s+1,k}/(e_{sk}+e_{s+1,k})=P(S=s|S=s-1\vee S=s,\Theta =k). \end{aligned}$$

The MLR property requires this last expression is non-decreasing in *k*. Hence, defining $\varvec{d}_s$ analogous to $\varvec{d}_i$, we define $V_{\text{ MLR }}$ as the average of $V_s$ obtained from (7) after substituting the item index by the sum score *s*.

**Fig. 10:**
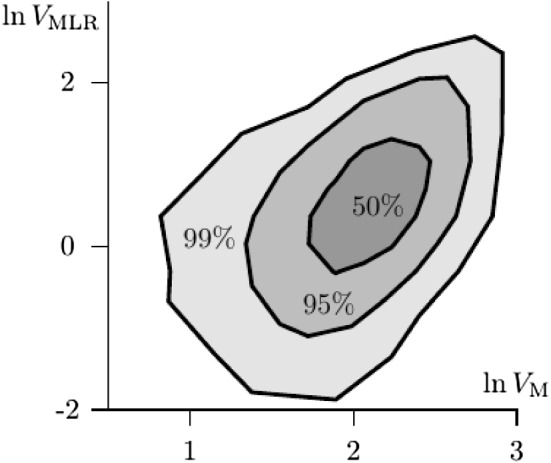
Empirical confidence regions of the size of the violation of M against the size of the violation of property MLR (on a logarithmic scale).

Figure 10 contains the density plot with the estimated 50%, 95%, and 99% confidence regions of $\ln V_{\text{ M }}$ and $\ln V_{\text{ MLR }}$, which shows a weak but positive relationship between the size of the violations of M and the size of the violations of MLR. As the size of the violation of M increases, so does the strength of the relationship. However, the size of the violations of MLR is generally small, with ${\overline{V}}_{\text{ MLR }}=2.011$ (the 1st and 3th quartile at 1.018 and 2.565, respectively). This means that none of the violations of M substantially invalidate the MLR property. The values $V_{\text{ MLR }}$ were further compared between the True and False cases, for each property. These results showed no difference beyond a small effect size for any of the observable properties. Hence, the results suggest that the MLR property is robust against violations of assumption M.

Molenaar (1997) generalized the MH model to polytomously scored items, where assumption M can be defined for different definitions of the response function (Mellenbergh, 1995). Unlike the MH model for binary response data, these polytomous models do not imply the MLR property (Hemker et al., 1996, 1997) without imposing additional restrictions on the shape of the response function (Ligtvoet, 2012). Although these polytomous models (assuming UD and LI) do not imply the MLR property, Van der Ark (2005) found that generally only few violations of MLR actually occurred, and that these violations had little effect on the ordering of respondents by their sum score. Our results for violations of M for binary response data are in line with these findings.

### Violations of Local Independence (Continued)

For the practical use of the sum score, it was found that the violations of M have little impact on the validity of the MLR property. Here, we consider again the MH model assumption of LI and investigate the impact a violation of LI has on the MLR property, using a latent class approach. As a model for generating the probabilities $P(\varvec{X}=\varvec{x}|\Theta =k)$, the assumptions proposed by Holland (1981) are considered (Sect. 1.1.5), for $J=3$ items. This small number of items clearly limits the extent to which the results can be generalized (as with the previous studies), so the results of this study should only be interpreted tentatively.

#### Procedure

For eight latent classes, let the matrix ${\mathbf {P}}=(\varvec{p}_1,\ldots ,\varvec{p}_8)$ contain the elements $p_{jk}=P(\varvec{X}=\varvec{x}|\Theta =k)$, for which assumption LND dictates that each $\varvec{p}_k$ is SPOD. For three items, SPOD coincides with property A, so LND implies that ${\mathbf {K}}\log ({\mathbf {M}}\varvec{p}_k)\ge \varvec{0}$, for $k=1,\ldots ,8$, and with the design matrix ${\mathbf {M}}$ given in (2). That is, LND imposed constraints on the entries within each column of ${\mathbf {P}}$. The monotonicity requirements in (4a) and (4a) impose additional constraints across the columns of ${\mathbf {P}}$. Let ${\mathbf {N}}_0=((0,1)^\prime ,(1,1)^\prime )$, ${\mathbf {N}}_1={\mathbf {N}}_0\otimes {\mathbf {N}}_0\otimes {\mathbf {N}}_0$, and ${\mathbf {N}}_2$ is like ${\mathbf {N}}_1$ but with its columns reversed. Then, the monotonicity assumption implies that the elements within each rows of ${\mathbf {N}}_1{\mathbf {P}}$ are non-decreasing in *k*, and for ${\mathbf {N}}_2{\mathbf {P}}$ non-increasing in *k*. Hence, the assumptions proposed by Holland (1981) correspond to the restrictions impose on ${\mathbf {P}}$ by the matrices ${\mathbf {M}}$, ${\mathbf {N}}_1$, and ${\mathbf {N}}_2$. Using the Gibbs sampler (Sect. 2.3), a total of 2000 such matrices ${\mathbf {P}}$ were simulated. Next, let ${\mathbf {E}}=\mathbf {FP}$, with element $e_{sk}=P(S=s-1|\Theta =k)$. Then, for each matrix ${\mathbf {P}}$ the statistic $V_{\text{ MLR }}$ can be computed (as above), with $V_{\text{ MLR }}$ expressing the size of the violation of the MLR property, as a result of relaxing the LI assumption.

#### Results

The results of the simulation yield the average ${\overline{V}}_{\text{ MLR }}=6.404$ (with the 1st and 3th quartile at 3.065 and 8.361, respectively). These violations of the MLR property are substantially higher than those found above due to the violations of the M assumption. Hence, the property of MLR is sensitive to violations of LI. Unfortunately, the assumption LI in our setup does not lend itself for an expression that can serve as a measure for quantifying the size of the violation of the LI assumption.

Evaluating the properties MM and CA (based on the previous analysis in Sect. 3.1), the results showed that property MM was satisfied for half or the cases, whereas CA was satisfied 37.5% of the time. Neither of the properties was found to be sensitive to the size of the violations $V_{\text{ MLR }}$.

## Discussion

Observable properties were evaluated that are all implied by the MH model for binary response data. Any violation of a property discredits the MH model assumptions. The most restrictive of these properties is CA, whereby each of the other properties can be interpreted as an incomplete test of CA. The incomplete tests of CA are hierarchically related and differ with respect to the inequality restrictions that they impose on the observable response distribution. The least restrictive of the properties is NC, and it implies that all covariances between pairs of item variables are nonnegative. The NC property forms the basis of the scalability coefficients used in Mokken scale analysis. The other incomplete tests of CA take into consideration the higher-order moments contained in the trivariate and multivariate distributions of the item scores.

The practical assessment of property CA is limited by the large number of inequality restrictions it imposes. These large number of inequality restrictions not only limit the feasibility of a global test of CA (as for property A), but for local (diagnostic) tests also induce problems associated with multiple testing. In addition to the large number of inequality restrictions, the assessment of the MH model assumptions will inevitably need to deal with sparse observations. Particularly the property of $\hbox {MTP}_2$, which pertains to the joint distribution of individual response patterns, is sensitive to such sparse observations, and as a result, to sampling error. Due to the number of restrictions imposed by CA and the problem of sparseness of observations, the practical assessment of the MH model assumptions always relies on an incomplete test of CA.

### Complexities of the Observable Properties

The computational burden associated with the large number of inequalities means that the observable properties could be studied only for small numbers of items. In a first series of small studies, we investigated the loss of information, when instead of CA an incomplete test of CA is used. For this purpose, the complexities of the incomplete tests were defined as their tendency to agree with a wide range of patters of data, with CA being the least and NC the most complex of the properties considered. For more than three items, the distinction between the complexities of CA and the incomplete tests of CA was found to be very large, and increased with increasing number of items. The exception to this rule was $\hbox {MTP}_2$, which agrees largely with CA. It may therefore be suggested that $\hbox {MTP}_2$ provides a practical alternative to CA for testing the MH model assumptions, which is associated with little loss of power.

Two remarks about the complexities of the properties are in order. First, the definition of complexity allowed for the loss of information to be studies, without relying on sample size, but this also means that we cannot infer from these results the exact extent to this loss of information translates to a loss of power when assessing the properties on real data. Second, psychological and educational tests contain items that are expected to relate to a common attribute, by design. Real response data will therefore generally agree more with the observable properties than random response patterns from a flat distribution. The complexities of the properties as presented here thus only provide a benchmark against which the relative agreement of different properties can be compared, when applied to real data. This is similar to the way the BIC penalizes the likelihood by the number of parameters. Here, the complexity, in terms of the number, also does not relate to real data.

### On the Sensitivity to Model Violations

A second series of studies was performed to investigate the sensitivity of the observable properties to different violations of the MH model assumptions M, LI, and UD. Only the properties CA and $\hbox {MTP}_2$ were found to be sensitive to violations of assumption M. However, these violations of M seem to have little impact on the MLR property for ordering respondents by means of their sum scores. The assumption of LI appears to be more relevant to the MLR property. Property CA was found to be sensitive to violations of LI (here, CA coincides with $\hbox {MTP}_2$), and to a lesser degree also MM. Finally, a violation of UD does not imply that $\hbox {MTP}_2$ is violated.

Besides the incomplete tests of CA considered, other observable properties have been proposed that were not considered. When assessing property MM in Mokken scale analysis, sparse observations are accounted for by joining adjacent rest scores into rest-score groups (Van der Ark, 2007). Assessing MM across these rest-score groups thus constitutes an incomplete test for MM. An incomplete test of CA can be similarly obtained by conditioning on the rest scores (Straat et al., 2016), or some other ‘carefully selected’ sub-test score as suggested by Stout (2002). Further, Ellis and Junker (1997) and Junker and Ellis (1997) provide a characterization of the MH model, whereby the vector of item variables is taken to be embedded within an infinite sequence of item variables (cf. Junker, 1991, 1993; Stout, 1987, 1990). Within this framework, other the observable properties have been proposed, like *vanishing conditional dependence* and *negative conditional covariance* (De Gooijer & Yuan, 2011; Junker, 1993; Yuan & Clarke, 2001).

### Implications

The results of the studies presented show that CA is a difficult property to assess. Most of the incomplete tests of CA are associated with a substantial loss of information and seem not to be sensitive to specific violations of the MH model assumptions. However, it is also good to keep in mind that any violation of any of the properties considered is sufficient for discrediting the MH model. The challenge herein lies in combining the multitude of information obtained from the data to derive at a single conclusion about the significance of observed violations. This problem can be illustrated in Fig. 3, which shows the results of the log-odds ratios related to CA. Here, only 78 restrictions were considered, but it is not obvious from the results how to combine these into a single conclusion about the validity of the MH model assumptions. A global test may produce a single *p*-value for this example, but becomes infeasible for more items. Also, different tests might balance the odds on the left and right differently or overemphasize the extreme values. These issues, however, mostly relate to goodness of fit. This is the other aspect of model selection that we didn’t focus on.

The primary focus of this paper is complexity, which mostly concerned the inferences that we can make about CA, based on an incomplete test. It is about the extent to which the confirmation of an incomplete test of CA warrants the validity of CA or (by extension) the MH model assumptions. The results of our analysis have specific implications for the interpretation of results of automated item selections procedures in Mokken scale analysis (Brusco et al., 2015; Mokken, 1971; Molenaar & Sijtsma, 2000; Sijtsma & Molenaar, 2002; Straat et al., 2013). As explained in Mokken et al. (1986, p. 280), the selection of items based on requirement imposed on the scalability coefficients provides an *operational definition* of a scale that need not necessarily agree with the MH model. Beside the issue of sampling error, our results show that rules of thumb used for construction such scales are rather arbitrary (cf. Hemker et al., 1995; Smits et al., 2012). In addition, in constructing these scales, the higher-order moments contained in the multivariate distributions of the item scores are ignored, which was shown to be associated with a substantial loss of information about the validity of the MH model assumptions. Hence, the scales produced by the automatic item selection procedure may not be very informative about the model underlying the scale and as such provide only an initial selection of items that require further analysis using more powerful tests for detecting violations of the model assumptions.

### Conclusion

The MH model is a very general model, which assumptions are shared by many of the response models used in practice. The assessment of these assumptions thus has implications that stretch beyond just the use of the MH model. As mentioned by Molenaar (2004), the inferences from a model are contingent on the validity of the model assumptions. A global test of goodness of fit may reject a model, but this would tell us little about why this is the case or what the problem might be. More research is required about the extent to which the assumptions and the specifications of response models influence the type of inferences one wishes to make (Sinharay & Haberman, 2014; Crişan et al., 2017). For example, our results suggest that the MLR property is less dependent on the specification of the item response functions (cf. Van der Ark, 2005) than on the LI assumption. This is important for the applied researcher who may want to test the MH model, not because she cases so much about the model, but because it allows respondents to be ordered on a common scales and it implies testable properties that reassure her that the decisions and inferences she makes based on the sum scores are theoretically justified and empirically supported.

## Appendix

*Assuming* $\varvec{p}>\varvec{0}$, *SPOD coincides with property A, in case of* $J=3$ *binary variables.*

For any subset of two variables from $\varvec{X}=(X_1,X_2,X_3)$, SPOD implies that the covariance between the two variables is positive. This corresponds to the first three rows of the matrix in (2) for the three distinct subsets $\varvec{V}=(X_2,X_3)$, $\varvec{V}=(X_1,X_3)$, and $\varvec{V}=(X_1,X_3)$, respectively. The remainder of the proof consists of going through the process of exhaustively listing all restrictions imposed by SPOD, and expressing these in terms of the log-odds ratios. It can then be shown that the last six rows of the matrix in (2) match one to one with those obtained for property SPOD. As an example, consider the inequality in (5c), which reduces for $\varvec{Y}=(X_1,X_2)$ and $\varvec{Z}=X_3$ to $(p_7+p_8)(p_1+p_3+p_5+p_7)\ge p_7$ and yields $\ln p_8-\ln (p_2+p_4+p_6)-\ln p_7+\ln (p_1+p_3+p_5)\ge 0$. The last inequality is obtained from (1) using the eighth row of the matrix in (2) for ${\mathbf {M}}$. The remaining five inequalities can be obtained similarly.

*Assuming* $\varvec{p}>\varvec{0}$, *MM and NC jointly imply the A, in case of* $J=3$ *binary variables.*

For property A, matrix (2) contains in its first three rows the constraints imposed by NC. Further, the first two rows of the matrix in (3) correspond to the MM property for $i=1$, which implies both $P(X_1=0,S=0)P(X_1=1,S>0)\ge P(X_1=1,S=0)P(X_1=0,S>0)$ and $P(X_1=1,S=2)P(X_1=0,S<2)\ge P(X_1=0,S=2)P(X_1=1,S<2)$. These last two inequalities correspond to the restrictions imposed by the fourth and fifth row of (2). Likewise, the remaining four restrictions in (3) imply the last four restrictions in (2).
